# Comparing the Performance of CMCC-BioClimInd and WorldClim Datasets in Predicting Global Invasive Plant Distributions

**DOI:** 10.3390/biology12050652

**Published:** 2023-04-26

**Authors:** Feixue Zhang, Chunjing Wang, Chunhui Zhang, Jizhong Wan

**Affiliations:** 1 State Key Laboratory of Plateau Ecology and Agriculture, Qinghai University, Xining 810016, China; 2 College of Agriculture and Animal Husbandry, Qinghai University, Xining 810016, China

**Keywords:** AUC, CMCC-BioClimInd, global scale, invasive plants, species distribution modelling, Maxent, WorldClim

## Abstract

**Simple Summary:**

The species distribution model has been widely used to predict the distribution of invasive plant species based on bioclimatic variables. However, the specific selection of bioclimate variables may affect the performance of the species distribution model. Here, we tested a new bioclimate variable dataset (i.e., CMCC BioClimInd) and used it in the species distribution model. We evaluated the predictive performance and explanatory power of WorldClim and CMCC-BioClimInd using AUC and omission rate, and also used the ODMAP protocol to record CMCC-BioClimInd to ensure reproducibility. The results indicate that CMCC BioClimInd can effectively simulate the distribution of invasive plant species. Based on the contribution rate of CMCC-BioClimInd to the distribution of invasive plant species, we inferred that the modified simplified continentality index and modified Kira warmth index from CMCC-BioClimInd had a strong explanatory power. Under the 35 bioclimatic variables of CMCC-BioClimInd, alien invasive plant species are mainly distributed in equatorial, tropical and subtropical regions. We tested a new bioclimate variable dataset to simulate the distribution of invasive plant species worldwide. Our research provides a new perspective for risk assessment and management of global invasive plant species.

**Abstract:**

Species distribution modeling (SDM) has been widely used to predict the distribution of invasive plant species based on bioclimatic variables. However, the specific selection of these variables may affect the performance of SDM. This investigation elucidates a new bioclimate variable dataset (i.e., CMCC-BioClimInd) for its use in SDM. The predictive performance of SDM that includes WorldClim and CMCC-BioClimInd was evaluated by AUC and omission rate and the explanatory power of both datasets was assessed by the jackknife method. Furthermore, the ODMAP protocol was used to record CMCC-BioClimInd to ensure reproducibility. The results indicated that CMCC-BioClimInd effectively simulates invasive plant species’ distribution. Based on the contribution rate of CMCC-BioClimInd to the distribution of invasive plant species, it was inferred that the modified and simplified continentality and Kira warmth index from CMCC-BioClimInd had a strong explanatory power. Under the 35 bioclimatic variables of CMCC-BioClimInd, alien invasive plant species are mainly distributed in equatorial, tropical, and subtropical regions. We tested a new bioclimate variable dataset to simulate the distribution of invasive plant species worldwide. This method has great potential to improve the efficiency of species distribution modeling, thereby providing a new perspective for risk assessment and management of global invasive plant species.

## 1. Introduction

Invasive plant species (IPS) are a global problem affecting agriculture, forestry, fisheries, human health, and natural ecosystems [1,2,3]. Climate change affects the identified IPS niche, thereby affecting their regional and global distributions [4,5]. The global average surface temperature has increased by about 0.6% in the past century [6,7]. The average climate model mainly represents the temperature and precipitation cycles within a year (month to season) and directly affects biological species’ distribution, abundance, and interaction [6,8]. Furthermore, climatic change has damaged the ecological balance [9,10]. Climate change affects invasive plants’ growth, development, physiological characteristics, and distribution pattern [1,5,6,11]. The growth and resistance of some species at the edge of the climate zone are weakened, and they will be more vulnerable to damage from invasive organisms [1,5,11]. Additionally, IPS often hinder local species from acquiring adequate nutrients, water, and light [12,13]. They also alter the invaded soils’ chemistry, hydrology, and water-holding capacity, thereby altering local plant community dynamics [14,15]. Therefore, reliable intrusion prediction methods are urgently required to effectively monitor and formulate reasonable management policies to reduce IPS spread, expansion, and risk [16,17,18,19,20,21]. Climate data sets have long been the basis for describing the native distribution of species [22] to solve problems based on protection [22,23,24,25]. Climate variables are also used to simulate invasive alien species’ potential distribution to better understand their evolution [21,26,27] and to assist biological control projects [19,28]. The global conformal data set is crucial to simulate the potential distribution of species in different continents [29].

Recently, multiple studies have outlined and compared species distribution modeling (SDM) methods [7,30]. SDM essentially predicts and describes the regional conditions suitable for species survival under climate change [31,32]. Environmental data especially climate data used for modeling, are among the least studied sources for SDM uncertainty [30]. With the increased availability of climate information, several global bioclimatic index datasets are being used as references by researchers [33,34]. WorldClim [29,35,36] is the most outstanding global climate dataset, often used in other applications, such as for ecological hydrology [37], comparisons of climatology [37,38], and the assessment of carbon stocks and their dynamics [39]. The WorldClim dataset includes 19 bioclimatic variables [40] calculated from monthly average temperature and total precipitation, including data from the global historical climate network dataset, which nearly meet distribution modeling requirements based on regression for most species [29].

The WorldClim dataset is valuable because of its refined resolution, including four spatial resolutions ranging from 30 s (~1 km^2^) to 10 min (~340 km^2^) [29,41]. However, it lacks the spatial details usually needed by models to evaluate impact, thereby fundamentally limiting its prediction for the essential spatial heterogeneity in steep mountain terrain [29,41]. In terms of spatial heterogeneity, a medium-resolution grid cell still spans the climate environment with an elevation difference of hundreds of meters [29,40]. Furthermore, it was also suggested that the 19 indicators of bioclimate variables were insufficient to simulate the distribution of plants and other organisms because plant growth requires more than precipitation and temperature [42]. For example, although there is a lot of precipitation in some areas, there is relatively increased evaporation; therefore, the water is transferred away from plants in different ways [43].

This paper describes the development and testing of a global dataset that can expand the availability of spatial information to regions [44,45,46] by testing a new indicator called the CMCC-BioClimInd [46], comprising 35 variables with a spatial resolution of 0.5°, including the historical period (1960–1999) and two future time ranges (2040–2079 and 2060–2099). The CMCC-BioClimInd data set is a collection of 11 CMIP5 climate simulations obtained by improving climate reanalysis. Research shows that the impact of climate change on the protection and management of wildlife, plants, and natural resources can be accurately estimated in different spatial ranges and research fields [46]. The CMCC-BioClimInd can more easily and quickly infer the relationship between the study’s subject and climate variables [47] and prevent the variability caused by the future trajectory and the physical properties of different indicators [48].

Noce et al. (2020) analyzed correlations between climate variables in two models (i.e., CMCC-BioClimInd and WorldClim) and the cause of their differences. They proved that CMCC-BioClimInd is accurate, comprehensive, and effective for predicting invasive species distributions and simulating climate change [46]. To our knowledge, no one has used CMCC-BioClimInd to predict invasive plant distributions nor applied it in practice. WorldClim is the most used dataset to predict species and potential invasive plant species distributions, which is greatly innovative (e.g., [2,15,32,49]).

Although species distribution models are widely used, the reproducibility of SDM methods is often limited due to the lack of reporting standards and the uncertainty of their predictions [7,30,31]. Therefore, here the ODMAP scheme was used to enhance the rationality and repeatability of this research [50,51]. The ODMAP (overview, data, model, evaluation, and prediction) reporting protocol provides a standardized way to communicate SDM results and outputs by describing objectives, model assumptions, scaling issues, data sources, model workflows, model predictions, and uncertainties [50,51]. The ODMAP protocol has two main purposes. First, it provides a checklist detailing the key steps of model construction and analysis to authors. Second, it introduces a standard documentation method to ensure transparency and repeatability [50,51]. Here, we tested the CMCC-BioClimInd dataset, described its basic elements, and detailed metadata based on the ODMAP (provided in Table S1).

We screened 11 most representative species of the 100 most dangerous alien invasive species in the world. We introduced a new global dataset of bioclimatic indicators to predict the distribution of invasive species and compared it with WorldClim (most used) to verify the prediction quality of the CMCC-BioClimInd data set and its results. This study specifically aimed to (a) compare the prediction performance of invasive species distributions by comparing WorldClim and CMCC-BioClimInd AUC values; (b) identify the most effective CMCC-BioClimInd variables affecting the distribution of invasive species; (c) determine the potential IPS distributions based on CMCC-BioClimInd; and (d) evaluate bioclimatic variables causing differences in IPS distributions for the same first 19 variables of WorldClim and CMCC-BioClimInd.

## 2. Materials and Methods

### 2.1. Occurrence Data 

**Figure 1:**
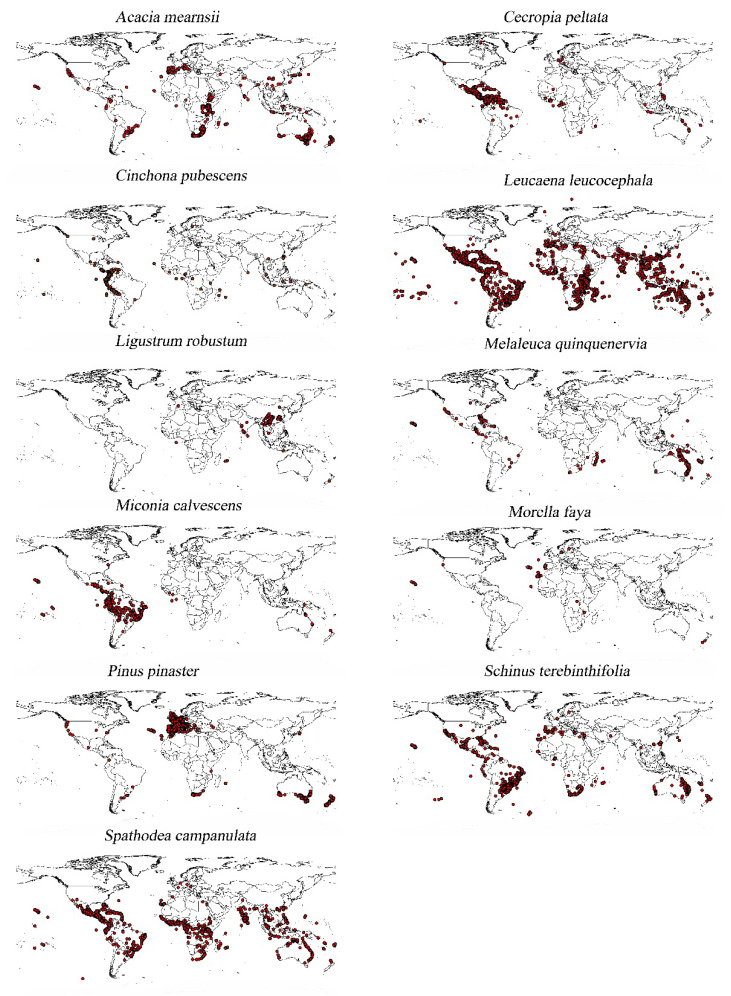
Geographical coordinates of the distribution of 11 IPS.

According to the expert group on invasive species, the world’s most invasive non-native species were compiled, among which the most important 11 IPS [52] were *Ligustrum robustum*, *Cinchona pubescens*, *Morella faya*, *Miconia calvescens*, *Cecropia peltate*, *Spathodea campanulata*, *Melaleuca quinquenervia*, *Schinus terebinthifolia*, *Acacia mearnsii*, *Leucaena leucocephala*, and *Pinus pinaster*. The occurrence records of these species were downloaded from the Global Biodiversity Information Facility (GBIF; https://www.gbif.org; https://doi.org/10.15468/dl.uxpqxy, https://doi.org/10.15468/dl.hrhg96, https://doi.org/10.15468/dl.9up5qj, https://doi.org/10.15468/dl.c2r3na, https://doi.org/10.15468/dl.qpgg5m, https://doi.org/10.15468/dl.fm3kec, https://doi.org/10.15468/dl.h77k3r, https://doi.org/10.15468/dl.8amej, https://doi.org/10.15468/dl.ftac38, https://doi.org/10.15468/dl.z84z4x and https://doi.org/10.15468/dl.h6h4fe (accessed on 2 April 2022); Figure 1). We downloaded species distribution data from 1970 to 1999 on GBIF, because only between 1970 and 1999 did the time periods of the two datasets coincide.The data we downloaded were processed as follows: (1) Carefully checked and screened the inaccurate or heteronymous species; (2) Deleted records with the same longitude and latitude; (3) Deleted duplicate records in a specific spatial resolution area [2,51]. Finally, a total of 390,000 geographical coordinate points of these 11 species were included in our analysis [53,54].

### 2.2. Data of Bioclimatic Variables

The origin of CMCC-BioClimInd is based on the daily time series of temperature and precipitation available in the weather data set of Water and European Medium-Range Weather Forecast Centre (ECMWF) reanalysis (ERA-40) [55], which is described in detail for the historical period (http://www.eu-watch.org/data_availability, accessed on 4 April 2022). The CMCC-BioClimInd data set is from (https://doi.org/10.1594/PANGAEA.904278, accessed on 5 April 2022; [46]). We obtained a set of 35 bioclimatic variables with a spatial resolution of 0.5° × 0.5° (1960–1999), covering the entire world (excluding Antarctica) [46]. WorldClim downloaded from (https://www.worldclim.org, accessed on 6 April 2022). A set of 5 arc minutes (10 × 10 km^2^) spatial resolution for 19 bioclimatic variables (1970–2000) [29,35,36] required the authors to resample the WorldClim climate variable to 0.5° resolution in order to be consistent with the resolution of CMCC-BioClimInd climate variable. The details of bioclimatic variables were shown in Table S2. 

We ran the Maxent model four times, namely, CMCC-BioClimInd, CMCC-BioClimInd (bio1–bio19), CMCC-BioClimInd (bio20–bio35), and WorldClim. By running it four times, we clearly compared CMCC-BioClimInd and WorldClim performance and verified the invasive species distribution prediction accuracy. Running CMCC-BioClimInd (bio1–bio19) compared the climate variable difference on invasive species distribution to WorldClim. Running CMCC-BioClimInd (bio20–bio35) revealed the impact of the new variables on invasive species distribution in addition to the first 19 variables, and more fully revealed the effectiveness of the new variables.

### 2.3. Modelling Approach and Evaluation

Based on species occurrence data and relevant environmental variables, the Maxent model is used to model species distribution under climate change [56,57]. Here, we established a logistic regression model with data from the 11 IPS distributions as response variables, and by running the Maxent model four times using the climate variables in the four climate data sets, namely, WorldClim and CMCC-BioClimInd (bio1–bio35), CMCC-BioClimInd (bio1–bio19), and CMCC-BioClimInd (bio20–bio35). The IPS distributional data were divided into a random training test set (auctest, 75%) and a test model set (auctrain, 25%). The regularization multiplier was set to two and the number of replicates to four [56,58,59].

We use the area under the curve (AUC) of the receiver’s operating characteristics to evaluate the prediction accuracy of the species distribution model. The AUC takes each value of the prediction result as a possible threshold, and then obtains the corresponding sensitivity and specificity values to calculate the curve [58]. The greater the AUC value, the greater the deviation between species distribution and random distribution (i.e., AUC = 0.5; [57,59]). The greater the correlation between variables and models, the higher the accuracy of the models. An AUC > 0.7 indicates that the model is effective [57]. We have added the omission rate test metric. The omission rate refers to the proportion of evaluation areas that are not within the scope of the model once converted to binary prediction [60,61]. The omission rate provides information about discrimination and overfitting evaluated under specific thresholds. Generally speaking, the lower the omission rate, the higher the performance [60,61]. We evaluate the performance of the model through the AUC and omission, which has a certain degree of scientific accuracy.

### 2.4. Effects of Bioclimatic Variables on Global Invasive Plant Species Distributions

Firstly, we used a jackknife method to assess bioclimatic variable contribution to the species’ distribution probability. The jackknife method output format showed the bioclimatic variables of each data set to the distribution probability, with values ranging from 0 (representing the smallest contribution) to 100% (representing the largest contribution) [61,62]. Secondly, we used an independent sample *t*-test [62] to compare the contribution rates of 19 bioclimatic variables in WorldClim (bio1–bio19) and CMCC-BioClimInd (bio1–bio19). We evaluated the difference of the average contribution rate of the first 19 bioclimatic variables to the distributions of IPS between the two models. Finally, after running Maxent, we generated ASCII files for both models (CCMC-BioClimInd and WorldClim). In GIS, we used mathematical analysis to subtract the WorldClim invasive plant distribution probability map from the CCMC-BioClimInd map [46]. We then obtained the distribution difference map for the 11 invasive plants. Positive values indicated that the predicted CMCC-BioClimInd distribution probability in a specific area was higher than WorldClim [46]. The opposite was true for negative values [46].

## 3. Results

### 3.1. Importance of Bioclimatic Variables for the Distribution of Invasive Species

Four different data sets were used, and the Maxent model was run four times. Their average AUC values were: CMCC-BioClimInd (bio20–bio35) > CMCC-BioClimInd (bio1–bio35) > CMCC-BioClimInd (bio1–bio19) > WorldClim (bio1–bio19) (Table 1). These four models performed well, with an average AUC above 0.940. Comparison of their average omission rates: CMCC-BioClimInd (bio20–bio35) < CMCC-BioClimInd (bio1–bio35) < CMCC-BioClimInd (bio1–bio19) < WorldClim (bio1–bio19). t\The omission rates of all four datasets were less than 0.15, indicating that the CMCC-BioClimInd dataset effectively simulates invasive species distribution (Table 1).

Among the 11 invasive plants, the average contribution of different climate variables, or different climate data sets, to the probability of invasive plants differed. First, we compared the average climate variable contribution rates for invasive plants between WorldClim (bio1–bio19) and CMCC-BioClimInd (bio1–bio19). In WorldClim, temperature seasonality (bio4), mean temperature of the coldest quarter (bio11), and isothermality (bio3) contributed more than 10% to the invasive species distribution (Table 2). In CMCC-BioClimInd (bio1–bio19), bio4 and annual mean temperature (bio1) had the greatest impact on invasive species distribution, with values of 23.9% and 14.131%, respectively (Table 2). Regardless of the model selection, bio4 was the most critical factor affecting invasive species (Table 2). Additionally, the average contribution rates of bio1, mean temperature of warmest quarter (bio10), and precipitation of the driest quarter (bio17) in predicting invasive species distribution probabilities in the WorldClim (bio1–bio19) and CMCC-BioClimInd (bio1–bio19) models differed significantly (*p* < 0.05; Table S3).

We used the complete CMCC-BioClimInd (bio1–bio35) to analyze the average contribution to the 11 invasive species. Surprisingly, the average contribution rate of 16 new variables (bio20–bio35) for invasive species distribution reached 56.732%, while the original 19 variables only reached 43.268% (Table 2). Here, the modified Kira warmth index (bio26), simplified continentality index (bio27), and bio4 all contributed more than 10% to invasive species distribution, including bio26 (13.883%), bio27 (12.322%), and bio4 (10.774%) (Table 2). To ensure the accuracy of these 16 variables for predicting the invasive species distribution and to make the results more intuitive, we ran CMCC-BioClimInd (bio20–bio35) separately. The results were consistent with the bioclimatic variable contribution rate of the complete CMCC-BioClimInd set. Moreover, bio26 and bio27 contributed markedly more to invasive species distribution (Table 2).

### 3.2. Distribution Probability of Invasive Species

**Figure 2:**
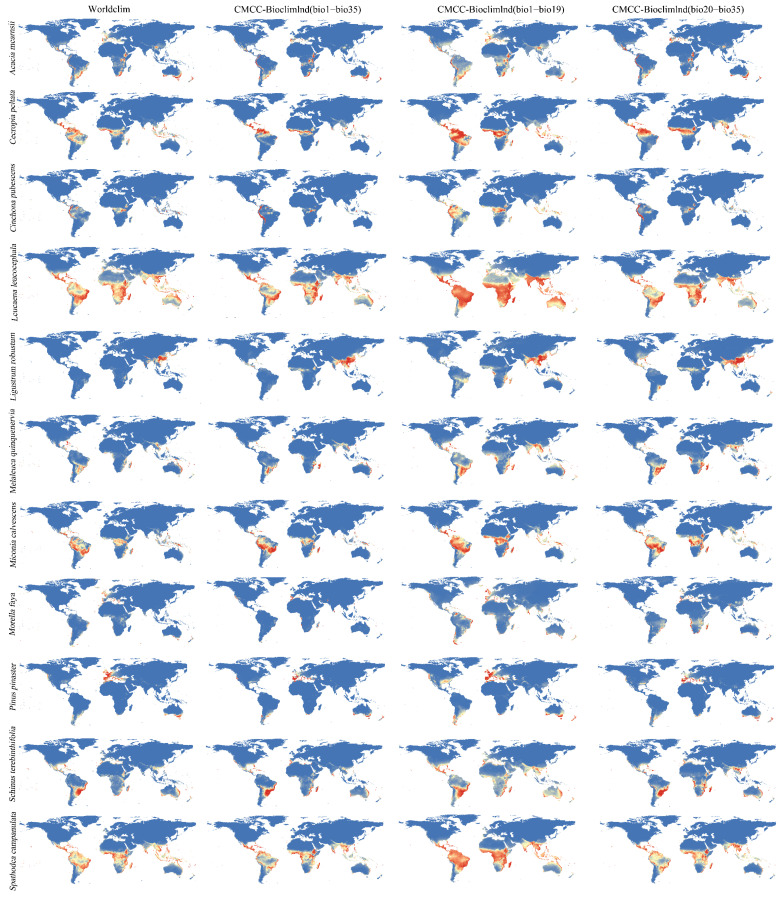
The impact of four model datasets (WorldClim, CMCC-BioclimInd bio1–bio35, CMCC-BioclimInd bio1–bio19, CMCC-BioclimInd bio20–bio35) on the distribution of 11 IPS. The distribution probability increases gradually from blue to red.

**Figure 3:**
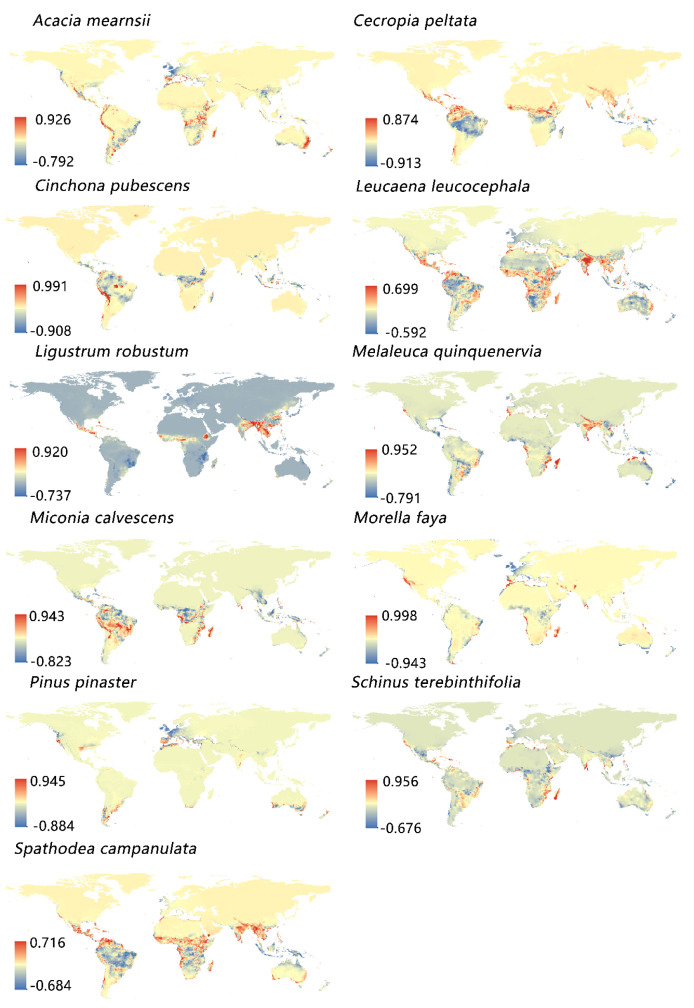
Maps of distribution probability differences calculated as CMCC-BioClimlnd minus WorldClim. Reddish tones indicate higher values based on CMCC-BioClimInd dataset in relation to the WorldClim dataset; bluish tones indicate the opposite.

The predicted species’ distribution ranges were roughly similar as assessed by running the distribution probability map of the Maxent model four times with different climate variables. They were also concentrated in the same region. However, they were not completely consistent (Figure 2). For these 11 species, the CMCC-BioClimInd (bio1–bio19) distribution ranges were significantly larger than WorldClim (bio1–bio19) (Figure 2). Moreover, the predicted climate variable distribution ranges of WorldClim were larger than those of CMCC-BioClimInd; however, the differences were small (Figure 1 and Figure 3). However, CMCC-BioClimInd and CMCC-BioClimInd (bio20–bio35) similarly predicted the invasive plant distributions and distribution probabilities (Figure 2).

The complete CMCC-BioClimInd set was considered the criterion since it was more comprehensive and accurate for invasive species prediction. *Acacia mearnsii* is distributed mainly in western and eastern South America, southern Australia, eastern Africa, and the western Mediterranean (Figure 2). *Cecropia peltata* is primarily located near the equator, especially in northern South America, with a high distribution probability (Figure 2). *Cinchona pubescens* is distributed mainly in western South America and central Africa, and the IPS main distribution range is also near the tropics (Figure 2). *Leucaena leucocephala* is widely distributed in the southern hemisphere, specifically in central Africa, Maldives, northern and southern South America, southern Asia, northeastern and western Australia (Figure 2). *Ligustrum robustum* is mainly distributed in southeast China, Southeast Asia, and eastern India. *Melaleuca quinquenervia* is distributed mainly near the Tropic of Capricorn and Cancer, with a small distribution range primarily concentric in the Maldives and Brazil (Figure 2). *Miconia calvescens* is distributed mainly between the equator and the Tropic of Cancer in Brazil, Peru, and central Africa (Figure 2). *Morella faya* showed a relatively scattered small distribution range (Figure 2) and only a few countries in the world are affected by it, being primarily distributed in southwest Spain, the Azores, Madeira, and the Canary Islands (Figure 2). *Pinus pinaster* is mainly distributed in the Mediterranean basin, southern Australia, and northeastern New Zealand, with a small distribution range (Figure 3). *Schinus terebinthifolia* is distributed near the Tropic of Cancer and the Tropic of Capricorn, mainly in eastern Brazil and eastern Africa (Figure 2). *Spathodea campanulata* is widely distributed and concentrated between the Tropic of Cancer and Capricorn, mainly in central Africa, north-central South America, southern Asia, and southern North America (Figure 2).

### 3.3. Differences in the Distribution Probability of Invasive Plant Species Predicted by the WorldClim and CMCC-BioClimInd Datasets

The differences in invasive plant distributions predicted by the two models for the 11 species were concentrated in the main distribution sites. Globally (except Antarctica), the invasive distribution probability maps predicted by the two models were roughly similar, and the area of difference was relatively small. The differences were concentrated in the Himalayas, Malaysia, and the Mediterranean (Figure 3). The species distribution probability predicted by CMCC-BioClimInd was greater than WorldClim in the Himalayas for *Leucaena leucocephala*, *Ligustrum robustum*, *Melaleuca quinquenervia*, and *Spathodea campanulate* (Figure 3). The distribution probability in Malaysia predicted by CMCC-BioClimInd was also higher than that of the WorldClim model for Acacia mearnsii, Leucaena leucocephala, *Melaleuca quinquenervia*, *Miconia calvescens*, *Morella faya*, *Schinus terebinthifolia*, and *Spathodea campanulata* (Figure 3). However, for *Acacia mearnsii*, *Morella faya*, and *Pinus pinaster*, WorldClim was higher than CMCC-BioClimInd when predicting the potential Mediterranean basin distribution (Figure 3).

## 4. Discussion

This investigation introduced a novel global bioclimatic index dataset [46] to predict the distribution of 11 invasive species. The 35 climate variables of CMCC-BioClimInd were divided into three datasets, and WorldClim was added to run the model. By comparing the AUC and omission rates of the four models, it revealed that the average AUC of all four models was higher than 0.94%. The omission rate in CMCC-BioClimInd was less than 0.2, while the omission rate in WorldClim was less than 0.25. Therefore, CMCC-BioClimInd was very effective and accurate in predicting the distribution probability for IPS (Table 1).

WorldClim’s climate dataset was derived by interpolating station data, while CMCC-BioClimInd’s was derived from climate reanalysis and 11 CMIP5 climate simulations [45,63]. The comparison revealed that the following variables contributed greatly to the invasive species distributions: bio4, bio11, and bio3 from WorldClim, and bio4 and bio1 from CMCC-BioClimInd (bio1–bio19) (Table 2). The literature indicated an increased correlation between bio3 and bio11, and the contribution rates of these two variables to invasive species distribution in WorldClim were very high [50,64] and inappropriate. Among the climate variables of WorldClim and CMCC-BioClimInd (bio1–bio19), the contribution rates of bio1, bio10, and bio17 to invasive species distribution were quite different (Table S3). Notably, the differences mentioned above were mainly in areas where the variable estimates were less accurate due to the paucity of ground observations [65], and some artifacts may have arisen from the interpolation function used to create the spatial gridded dataset [46]. The possible explanation for the observed differences between WorldClim and CMCC-BioClimInd, might be the different weights given to observations concerning climate modeling data when creating the datasets [46].

CMCC-BioClimInd has 35 environmental variables [46,66]; it was noted that the first 19 CMCC-BioClimInd climate variables were similar to WorldClim, and had 16 additional climate variables compared to WorldClim [46]. Our results showed that among the 35 environmental variables, bio26, bio27, and bio4 had a higher contribution to invasive species distribution (Table 2). Furthermore, when the CMCC-BioClimInd (bio20–bio35) variables were compared with CMCC-BioClimInd (bio1–bio19) variables, it was found that the latter 16 variables dominated the invasive species distribution probabilities, with a 57% contribution rate, while the first 19 variables only contributed about 43% (Table 2). In CMCC-BioClimInd, bio27 and bio26 contributed significantly to invasive species distributions (Table 2), inferring the importance of the last 16 variables to invasive species distributions. 

Although temperature and precipitation had different effects on invasive species distributions, inconsistent with previous studies [2,3], it was found that bio27 and bio26 had the most obvious effect on the distribution of invasive species (Table 2). Apart from how solar radiation varies with latitude, research has revealed that continentality is the most important factor controlling locality variation in Earth’s climate and affecting plant growth [67]. Generally, various Earth’s surface factors influence radiation fluxes, heat, and moisture at the air–land and air–water interfaces; these affect weather aspects such as temperature, precipitation, and cloudiness [67,68,69]. Additionally, previous studies have proved that the plant growth of the forest community is affected by the thermal climate to some extent; for example, there is a tendency for increased aboveground plant height, plant biomass, and the degree of forest canopy multi-layering toward warmer regions [70]. On the other hand, the diversity of component plant flora is extremely sensitive to changes in the thermal climate [70,71,72]. Plant growth requires more than just precipitation and temperature [73]. For example, studies have shown that although some areas receive sufficient precipitation and light, many factors, such as a high evaporation rate, serious water loss, and radiation, may result in poor species growth [43,49]. 

We consider that CMCC-BioClimInd can predict invasive plant distributions more comprehensively and accurately [46,73]. Other studies revealed that environmental variables were inconsistent for predicting species distributions [74,75]. The contribution rates of various species were different, associated with species habitat and to a certain extent, the mutual restriction between organisms [73]. For example, Dingle et al. (2000) found that annual rainfall and soil moisture explained 90% and 62% of migratory butterfly species richness in the Australian drought center, respectively [76]. However, these two factors were not significant for butterflies in the rainy areas of eastern Australia [76]. In eastern Australia, temperature seasonality has become the best single climate predictor of butterfly species richness [76].

It was also observed that the 11 IPS mainly distributed in tropical rainforests and grasslands, subtropical evergreen broad-leaved forests, and the Mediterranean region (Figure 2). These climate regions have abundant species resources, sufficient rain, heat, and forest resources [77]. Biodiversity is also richer in places with abundant plant resources [77]. For example, the invasive *Morella faya* is highly scattered throughout a narrow distribution area [78]. The introduction of fruit-eating birds promoted the spread of this fast-growing plant, and these plants quickly formed dense stands, endangering local plant growth [78].

Temperature and precipitation are essential for IPS biology [2,3,4,5]. On a large spatial scale, tolerance of invasive plants is usually linked with climate and the main habitat [6,75]. With climate change, invasive species from adjacent areas may cross national borders and become new biota elements [2,3]. Invasive species threaten plant growth in local habitats by competing with local vegetation, replacing grassland communities, reducing local biodiversity, and increasing water loss in riparian zones [4,13]. Therefore, CMCC-BioClimInd can predict potential alterations for invasive species with respect to future climate according to the 35 climate variables and help to implement timely preventive measures [46]. Otherwise, invasive plants’ economic loss and negative impacts on food security, biodiversity, and ecosystem services may soon sharply increase [1,77].

We found a significant difference in the response of IPS distribution probabilities between CMCC-BioClimInd and WorldClim near the Himalayas. According to the literature, in the Himalayas, the temperature and precipitation in CMCC-BioClimInd were higher than in WorldClim [46]. Therefore, the invasive plant distribution probabilities based on CMCC-BioClimInd were also higher in the Himalayas. Notably, a large distribution probability difference exists near the equator, Mediterranean, Malaysia, and western and eastern Australia (Figure 3). The effect of climate data’s quality may also affect species distribution modelling [49]. To predict a reasonable future distribution, the indispensable bioclimatic variables used in a species distribution model must be reliable [49]. We have the following conjectures about the differences caused by the two data sets on the distribution of invasive species: (1) It may be because the CMCC-BioClimInd data set has 16 additional climate variables with greater impact compared to WorldClim for the distribution of invasive species [35,46]; (2) For bioclimatic variables, the sources of these variables are different [35,36,46]. Coarse-scale bioclimatic information may be insufficient or inconsistent for species distribution models derived from finer-scale species occurrence data [49]; (3) This may be because the distribution area for the main invasive species was in the region where the variable estimates were less accurate due to the paucity of ground observations. Furthermore, the observed differences between WorldClim and CMCC-BioClimInd might be because of the different observation’s weights used with respect to climate modeling data when creating the datasets [35,46]. To limit IPS damage to global biodiversity, safety, and the economy, effective measures must be taken to prevent their further expansion [2,4,5]. Our research also provides a new global IPS risk assessment and management perspective.

Our research also has some limitations. (1) To some extent, the multiple linearities between variables in the CMCC-BioClimInd and WorldClim dataset were not addressed. Research has shown that correlation analysis refers to comparing and analyzing two or more related variables to measure the degree of correlation between variables [79]. There are various data indicators, and most overlap with each other, resulting in large redundancy in the data. Using a wide range of similar data cannot yield comprehensive information, resulting in a phenomenon where the amount of data and information is not proportional and potentially serious fitting [80,81]. In the selection and determination of MaxEnt environmental variables, the number of environmental variables can be changed due to their different abilities to determine species distributions [82]. The number of environmental variables largely affects MaxEnt’s ability to simulate the distribution of invasive plants by altering the model’s complexity [82]. Therefore, in future research, the multicollinearity problem between variables should be addressed and environmental variables that are the most important for studying species distribution should be selected. (2) In this study, the number of replicates set in the species distribution model was too low. For example, recent research on species distribution modelling has shown that MaxEnt often produces better classification results when users choose the optimal parameters [83]. Research results indicate that model parameterization significantly impacts the prediction accuracy of MaxEnt; therefore, appropriate parameterization is highly correlated with good classification results [84]. Thus, in future research, the number of iterations for the Maxent model will be set to 10–100. (3) Using AUC and commission rates to determine MaxEnt’s modeling performance does not provide the best model predictions. With imbalanced datasets, AUC may be misleading as the number of positive and negative samples is uneven. In this case, AUC may have overestimated the classifier’s performance. In addition, the score of AUC ignores the actual probability value, making it insensitive to changes in the predicted probability of maintaining its ranking, and the testing performance of ROC in spatial regions is rarely successful [85,86]. Additional performance evaluation indicators (e.g., TSS, Kappa, the null model for significance testing, Boyce index, Still block cross-validation) should be included in future studies [87,88,89,90]. (4) Although MaxEnt is widely used to simulate plant invasion [91], research has shown that the number of species recorded, the number of environmental variables, and the spatial scale all affect the performance of the MaxEnt distribution model, indicating that these three inputs can lead to uncertainty in the invasive plant MaxEnt [80]. In future research, we can use models such as Maxlike and general linear models to evaluate invasive plants.

## 5. Conclusions

The CMCC-BioClimInd datasets improve existing global bioclimatic datasets used for SDM. This is a pragmatic compromise that addresses some of the limitations of the currently available products and is accurate for predicting invasive species distributions. In the rapidly changing global environment, bioclimatic species modelling has become an important tool for answering many conservation biology and invasion ecology questions. The CMCC-BioClimInd dataset can provide a wide range of core functions for these models. After combining 35 CMCC-BioClimInd climates, bio27 and bio26 were found to greatly impact invasive species distributions. Furthermore, it was revealed that the invasive species were mainly distributed in areas with sufficient rain and heat, such as tropical rainforests and grasslands, and subtropical evergreen broad-leaved forests. Therefore, policymakers must reinforce the management of areas vulnerable to the six kinds of invasion and formulate effective strategies to prevent invasive plant expansion.

## Figures and Tables

**Table 1:** AUC values and omission rate of four climate data sets for modelling 11 invasive plant species distributions based on MaxEnt SDM. OR:omission rate; CMCC: CMCC-BioClimInd.

Species	WorldClim	OR	CMCC	OR	CMCC	OR	CMCC	OR
			(bio1–bio19)		(bio1–bio35)		(bio20–bio35)	
*Acacia mearnsii*	0.953	0.102	0.963	0.069	0.985	0.052	0.984	0.051
*Cecropia peltata*	0.954	0.090	0.961	0.059	0.979	0.042	0.980	0.043
*Cinchona pubescens*	0.969	0.081	0.977	0.054	0.966	0.055	0.972	0.056
*Leucaena leucocephala*	0.861	0.213	0.892	0.164	0.958	0.079	0.956	0.081
*Ligustrum robustum*	0.980	0.052	0.974	0.054	0.980	0.050	0.977	0.049
*Melaleuca quinquenervia*	0.967	0.082	0.967	0.059	0.972	0.056	0.975	0.054
*Miconia calvescens*	0.953	0.099	0.963	0.063	0.978	0.041	0.975	0.044
*Morella faya*	0.977	0.075	0.927	0.124	0.863	0.162	0.927	0.124
*Pinus pinaster*	0.962	0.073	0.955	0.068	0.976	0.046	0.976	0.046
*Schinus terebinthifolia*	0.941	0.116	0.936	0.100	0.963	0.076	0.962	0.074
*Spathodea campanulata*	0.913	0.160	0.944	0.095	0.975	0.055	0.974	0.057
*Average of all species*	0.948	0.104	0.951	0.083	0.963	0.065	0.969	0.062

**Table 2:** Average contribution of climatic variables from four climate datasets to the invasive plant species distributions across 11 species. Bold values mean a large contribution to the invasive plant species distributions.

Climate Variables	WorldClim (%)	CMCC-Bioclimlnd bio1–bio19 (%)	CMCC-Bioclimlnd bio1–bio35 (%)	CMCC-Bioclimlnd bio20–bio35 (%)
bio1	5.412	**14.131**	2.758	
bio2	2.477	1.819	3.113	
bio3	12.991	7.391	2.776	
bio4	**20.472**	**23.900**	**10.774**	
bio5	1.406	1.223	0.094	
bio6	7.112	4.134	0.130	
bio7	2.877	3.721	1.736	
bio8	1.021	0.553	0.023	
bio9	0.225	0.147	0.001	
bio10	0.556	4.836	1.878	
bio11	14.020	3.116	1.189	
bio12	5.255	3.440	0.657	
bio13	2.541	1.652	0.484	
bio14	3.721	5.371	2.726	
bio15	1.944	4.471	2.554	
bio16	4.865	8.741	1.140	
bio17	0.401	3.025	2.390	
bio18	5.544	2.950	3.433	
bio19	7.161	5.381	5.411	
bio20			1.004	1.590
bio21			1.293	5.250
bio22			0.041	2.386
bio23			3.910	7.617
bio24			4.795	8.041
bio25			9.401	10.085
bio26			**13.883**	**16.000**
bio27			**12.322**	**28.726**
bio28			0.500	2.042
bio29			0.279	1.939
bio30			0.129	0.757
bio31			0.198	0.692
bio32			0.054	0.176
bio33			5.633	6.748
bio34			0.793	4.807
bio35			2.497	3.143

## Data Availability

The data that support the findings of this study are available from the corresponding author upon reasonable request.

## Supplementary Materials

The following supporting information can be downloaded at: https://www.mdpi.com/article/10.3390/biology12050652/s1, Table S1: Description of the CMCC-BioClimInd dataset according to the ODMAP protocol; Table S2: Climate variables in CMCC-Bioclimlnd dataset including codes, full names and unIPS. bio1~bio19 were the same as WorldClim dataset; Table S3: The significance of differences in the average contribution rate (%) of bioclimatic variables to species distribution probability between WorldClim and CMCC-Bioclimlnd based on independent-sample *t* test.
