# Supplementary material for: Comparing the Performance of CMCC-BioClimInd and WorldClim Datasets in Predicting Global Invasive Plant Distributions
Source: Biology (Basel). 2023 Apr 26;12(5):652. doi: 10.3390/biology12050652 (PMC10215630; doi:10.3390/biology12050652)
Supplement: Supplementary file 1 [file biology-12-00652-s001.zip › biology-2315178-supplementary.pdf]

Supporting Materials

Table S1. Description of the CMCC-BioClimInd dataset according to the ODMAP protocol.

Section	Subsection	Element	Value
Overview	Authorship	Study title	CMCC-BioClimInd
Overview	Authorship	Author names	Feixue Zhang，Chun-Jing Wang, Chun-Hui Zhang1, Ji-Zhong Wan
Overview	Authorship	Contact	Zfx660109@163.com
Overview	Authorship	Study link	https://www.nature.com/articles/s41597-020-00726-5 (accessed on 2 April 2023)
Overview	Model objective	Model objective	Forecast and transfer
Overview	Model objective	Target output	The probability of distribution of invasive alien plants
Overview	Focal Taxon	Focal Taxon	species
Overview	Location	Location	the whole world
Overview	Scale of Analysis	Spatial extent	-180, 180, -90, 90 (xmin, xmax, ymin, ymax)
Overview	Scale of Analysis	Spatial resolution	0.5°
Overview	Scale of Analysis	Temporal extent	1970–1999
Overview	Scale of Analysis	Temporal resolution	We obtained a set of 35 bioclimatic variables with a spatial resolution of 0.5 ° × 0.5 ° (1960 – 1999), covering the entire world (excluding Antarctica) [46]. WorldClim downloaded from (https://www.worldclim.org). A set of 5 arc minutes (10 × 10 km ?) spatial resolution for 19 bioclimatic variables (1970 – 2000)
Overview	Scale of Analysis	Boundary	natural
Overview	Biodiversity data	Observation type	standardized momitoring
Overview	Biodiversity data	Response data type	presence/absence
Overview	Predictors	Predictor types	climatic
Overview	Hypotheses	Hypotheses	Climate variables affect the distribution of species
Overview	Assumptions	Model assumptions	The CMCC BioClimInd dataset has good performance
Overview	Algorithms	Modelling techniques	maxent
Overview	Algorithms	Model complexity	The invasive plant species distributional data were divided into a random training test set (auctest, 75%) and a test model set (auctrain, 25%). The regularisation multiplier was set to two and the number of replicates to four.
Overview	Algorithms	Model averaging	we employed two threshold-dependent measures: omission rates based on two threshold rules (10% calibration omission rate and lowest presence threshold, LPT = 0% calibration omission rate =minimum

training presence threshold of Maxent software). Omission rates are the proportion of evaluation localities that fall outside of the model once converted to a binary prediction. Omission rates provide information regarding both discriminatory ability and overfitting, evaluated at particular thresholds. In general, lower omission indicates higher performance (better discrimination between suitable versus unsuitable areas). In addition, overfit models have omission rates higher than theoretical expectations. The lowest presence threshold sets the threshold at the lowest value of the prediction for any pixel that contains a calibration locality and has an expected omission rate of zero for evaluation localities. Similarly, the 10% calibration omission rate rule sets the threshold at a value that excludes the 10% of calibration localities with lowest predictionand has an expected omission rate of 0.10. We obtained the two threshold-dependent measures and averaged their values as for AUC.

Based on species occurrence data and relevant environmental variables, Maxent model is used to model species distribution under climate change [56,57]. Here, we es-tablished a logistic regression model with data from the 11 invasive plant species distributions as response variables, and by running the Maxent model four times using the climate variables in the four climate data sets, namely, WorldClim and CMCC-BioClimInd (bio1-bio35), CMCC-BioClimInd (bio1-bio19), and CMCC-BioClimInd (bio20-bio35). The IPS distributional data were divided into a random training test set (auctest, 75%) and a test model set (auctrain, 25%). The regularisation multiplier was set to two and the number of replicates to fou.

Overview	Workflow	Model workflow

We use the area under the curve (AUC) of the receiver's operating characteristics to evaluate the

			prediction accuracy of the species distribution model. AUC takes each value of the prediction result as a possible threshold, and then obtains the corresponding sensitivity and specificity values to calculate the curve [60]. The greater the AUC value, the greater the deviation between species distribution and random distribution (i.e., AUC = 0.5). The greater the correlation between variables and models, the higher the accuracy of models. AUC > 0.7 indicates that the model is effective.we employed two threshold-dependent measures: omission rates based on two threshold rules (10% calibration omission rate and lowest presence threshold, LPT = 0% calibration omission rate = minimum training presence threshold of Maxent software). Omission rates are the proportion of evaluation localities that fall outside of the model once converted to a binary prediction. Omission rates provide information regarding both discriminatory ability and overfitting, evaluated at particular thresholds. In general, lower omission indicates higher performance (better discrimination between suitable versus unsuitable areas). In addition, overfit models have omission rates higher than theoretical expectations. The lowest presence threshold sets the threshold at the lowest value of the prediction for any pixel that contains a calibration locality and has an expected omission rate of zero for evaluation localities. Similarly, the 10% calibration omission rate rule sets the threshold at a value that excludes the 10% of calibration localities with lowest predictionand has an expected omission rate of 0.10. We obtained the two threshold-dependent measures and averaged their values as for AUC.
Overview	Software	Software	Maxent version 3.3.3
Overview	Software	Code availability	reinforcement-learning-kr/lets-do-irl
Overview	Software	Data availability	https://www.gbif.org/

Data	Biodiversity data	Taxon names	Ligustrum robustum, Cinchona pubescens, Morella faya, Miconia calvescens, Cecropia peltate, Spathodea campanulata, Melaleuca quinquenervia, Schinus terebinthifolia, Acacia mearnsii, Leucaena leucocephala, and Pinus pinaster
Data	Biodiversity data	Taxonomic reference system	*no have*
Data	Biodiversity data	Ecological level	Species-level
Data	Biodiversity data	Data sources	GBIF; https://www.gbif.org/
Data	Biodiversity data	Sampling design	temporal design
Data	Biodiversity data	Sample size	prevalence
Data	Biodiversity data	Clipping	no have
Data	Biodiversity data	Scaling	spatial and temporal thinning
Data	Biodiversity data	Cleaning	outlier presence/treatment
Data	Biodiversity data	Absence data	no have
Data	Biodiversity data	Background data	spatial and temporal buffer, bias correction
Data	Biodiversity data	Errors and biases	geo-referencing errors, sampling bias
Data	Data partitioning	Training data	training test set (auctest, 75%)
Data	Data partitioning	Validation data	cross-validation method
Data	Data partitioning	Test data	test model set (auctrain, 25%)
Data	Predictor variables	Predictor variables	Worldclim(bio1-bio19);CMCC-BioClimInd
Data	Predictor variables	Data sources	CMCC-BioClimInd 1.0 and WorldClim 2.0 datasets for the historical period
Data	Predictor variables	Spatial extent	-180, 180, -90, 90 (xmin, xmax, ymin, ymax)
Data	Predictor variables	Spatial resolution	0.5°
Data	Predictor variables	Coordinate reference system	ESRI PE string
Data	Predictor variables	Temporal extent	no have
Data	Predictor variables	Temporal resolution	no have
Data	Predictor variables	Data processing	pscaling/downscaling
Data	Predictor variables	Errors and biases	no have
Data	Predictor variables	Dimension reduction	no have
Data	Transfer data	Data sources	no have
Data	Transfer data	Spatial extent	-180, 180, -90, 90 (xmin, xmax, ymin, ymax)
Data	Transfer data	Spatial resolution	no have
Data	Transfer data	Temporal extent	no have
Data	Transfer data	Temporal resolution	no have

Data	Transfer data	Models and scenarios	Maxent
Data	Transfer data	Data processing	no have
Data	Transfer data	Quantification of Novelty	distance to training data
Model	Variable pre-selection	Variable pre-selection	Climate variables
Model	Multicollinearity	Multicollinearity	Not used
Model	Model settings	Model settings (fitting)	maxent: no settings provided
Model	Model settings	Model settings (extrapolation)	clamping
Model	Model estimates	Coefficients	median or mean posterior
Model	Model estimates	Parameter uncertainty	resampling
Model	Model estimates	Variable importance	contribution;jackknife
Model	Model selection - model averaging - ensembles	Model selection	information-theoretic approach for variable selection, shrinkage and regularization
Model	Model selection - model averaging - ensembles	Model averaging	derivation of weights
Model	Model selection - model averaging - ensembles	Model ensembles	model classes, model parameters, boundary conditions
Model	Analysis and Correction of non-independence	Spatial autocorrelation	Not used
Model	Analysis and Correction of non-independence	Temporal autocorrelation	Not used
Model	Analysis and Correction of non-independence	Nested data	fixed and random effects
Model	Threshold selection	Threshold selection	transforming continuous predictions into binary predictions
Assessment	Performance statistics	Performance on training data	AUC; False negative rate; False positive rate
Assessment	Performance statistics	Performance on validation data	AUC; False negative rate; False positive rate
Assessment	Performance statistics	Performance on test data	AUC; False negative rate; False positive rate
Assessment	Plausibility check	Response shapes	partial response plots
Assessment	Plausibility check	Expert judgement	map display
Prediction	Prediction output	Prediction unit	Species distribution probability
Prediction	Prediction output	Post-processing	clipping
Prediction	Uncertainty quantification	Algorithmic uncertainty	Setting parameters for the Maxent model
Prediction	Uncertainty quantification	Input data uncertainty	No have
Prediction	Uncertainty quantification	Parameter uncertainty	No have

Prediction	Uncertainty quantification	Scenario uncertainty	climate models
Prediction	Uncertainty quantification	Novel environments	masking

Table S2. Climate variables in CMCC-Bioclimlnd dataset including codes, full names and units for IPS. bio1~bio19 were the same as WorldClim dataset [46].

Code	Name	Unit
bio1	Annual mean temperature	°C
bio2	Mean diurnal range	°C
bio3	Isothermality	%
bio4	Temperature seasonality	°C
bio5	Max temperature of warmest month	°C
bio6	Min temperature of coldest month	°C
bio7	Temperature annual range	°C
bio8	Mean temperature of wettest quarter	°C
bio9	Mean temperature of driest quarter	°C
bio10	Mean temperature of warmest quarter	°C
bio11	Mean temperature of coldest quarter	°C
bio12	Annual precipitation	mm
bio13	Precipitation of wettest month	mm
bio14	Precipitation of driest month	mm
bio15	Precipitation seasonality	%
bio16	Precipitation of wettest quarter	mm
bio17	Precipitation of driest quarter	mm
bio18	Precipitation of warmest quarter	mm
bio19	Precipitation of coldest quarter	mm
bio20	Ellenberg quotient	°C/mm
bio21	Yearly positive temperature	°C
bio22	Sum of annual temperature	°C
bio23	Ombrotermic index	mm/°C
bio24	Yearly positive precipitation	mm
bio25	Modified Kira coldness index	°C
bio26	Modified Kira warmth index	°C
bio27	Simplified continentality index	°C
bio28	Mean temperature of warmest month	°C
bio29	Mean temperature of coldest month	°C
bio30	Mean temperature of driest month	°C
bio31	Mean temperature of wettest month	°C
bio32	Modified Thermicity index	°C
bio33	Ombrothermic index of summer and the previous month	mm/°C
bio34	Potential Evapotranspiration Hargreaves	mm
bio35	Potential Evapotranspiration Thornthwaite	mm

Table S3. The significance of differences in the average contribution rate (%) of bioclimatic variables to species distribution probability between WorldClim and CMCC-Bioclimlnd based on independent-sample t test.

Climate	WorldClim	CMCC-Bioclimlnd	*p*
bio1	5.412	14.131	0.022
bio2	2.477	1.819	0.649
bio3	12.991	7.391	0.188

bio4	20.472	23.900	0.724
bio5	1.406	1.223	0.762
bio6	7.112	4.134	0.407
bio7	2.877	3.721	0.795
bio8	1.021	0.553	0.458
bio9	0.225	0.147	0.571
bio10	0.556	4.836	0.000
bio11	14.020	3.116	0.094
bio12	5.255	3.440	0.464
bio13	2.541	1.652	0.617
bio14	3.721	5.371	0.294
bio15	1.944	4.471	0.055
bio16	4.865	8.741	0.351
bio17	0.401	3.025	0.000
bio18	5.544	2.950	0.260
bio19	7.161	5.381	0.652

Red: $p < 0.05$.
